# Cyto-Histological Profile of MicroRNAs as Diagnostic Biomarkers in Differentiated Thyroid Carcinomas

**DOI:** 10.3390/genes15030389

**Published:** 2024-03-21

**Authors:** Maria de Lurdes Matos, Mafalda Pinto, Marta Alves, Sule Canberk, Ana Gonçalves, Maria João Bugalho, Ana Luísa Papoila, Paula Soares

**Affiliations:** 1 Department of Endocrinology, Diabetes and Metabolism, Hospital Curry Cabral, Unidade Saúde Local São José, Centro Clínico e Académico de Lisboa, 1050-166 Lisbon, Portugal; 2 Institute for Research & Innovation in Health (i3S), Instituto de Patologia e Imunologia Molecular da Universidade do Porto (IPATIMUP), 4200-135 Porto, Portugal; mafaldap@ipatimup.pt (M.P.); scanberk@ipatimup.pt (S.C.); 3 Gabinete de Estatística do Centro de Investigação, Unidade Saúde Local São José, Nova Medical School, Centro de Estatística e Aplicações da Universidade de Lisboa (CEAUL), 1169-166 Lisbon, Portugal; marta.l.alves@gmail.com (M.A.); ana.papoila@nms.unl.pt (A.L.P.); 4 Department of Pathology, Centro Hospitalar Universitário de São João, 4200-135 Porto, Portugal; u018610@chsj.min-saude.pt; 5 Department of Endocrinology, Hospital de Santa Maria, Unidade Saúde Local Santa Maria and Medical Faculty, University of Lisbon, 1069-028 Lisbon, Portugal; maria.bugalho@chln.min-saude.pt; 6 Department of Pathology, Medical Faculty, University of Porto, 4200-135 Porto, Portugal

**Keywords:** microRNAs, miR-146b, miR-221, miR-222, miR-15a, differentiated thyroid carcinoma, papillary thyroid carcinoma, genetic mutations, *TERTp*, *BRAF*, *RAS*

## Abstract

Introduction: The repertoire of microRNAs (miRNAs) in thyroid carcinomas starts to be elucidated. Among differentiated thyroid carcinomas (DTCs), papillary thyroid carcinoma (PTC) is the most frequent. The assessment of miRNAs expression may contribute to refine the pre-surgical diagnosis in order to obtain a personalized and more effective treatment for patients. Aims: This study aims to evaluate (1) the miRNAs in a series of DTCs, and their association with the presence of selected genetic mutations in order to improve diagnosis and predict the biologic behavior of DTC/PTC. (2) The reliability of molecular tests in Ultrasound-guided Fine Needle Aspiration Cytology (US-FNAC) for a more precise preoperative diagnosis. Material and Methods: This series includes 176 samples (98 cytology and 78 histology samples) obtained from 106 patients submitted to surgery, including 13 benign lesions (controls) and 93 DTCs (cases). The microRNA expression was assessed for miR-146b, miR-221, miR-222, and miR-15a through quantitative reverse transcriptase-polymerase chain reaction (qRT-PCR). The results were analyzed by the 2^−ΔΔCT^ method, using miR16 as an endogenous control. Regarding PTC diagnosis, the discriminative ability of miRNAs expression was assessed by the area under the Receiver Operating Characteristic Curve (AUC). In PTCs, the association of miRNAs expression, clinicopathological features, and genetic mutations (*BRAF*, *RAS,* and *TERTp*) was evaluated. Results/Discussion: All the analyzed miRNAs presented a tendency to be overexpressed in DTCs/PTCs when compared with benign lesions, both in cytology and histology samples. In cytology, miRNAs expression levels were higher in malignant tumors than in benign tumors. In histology, the discriminative abilities regarding PTC diagnosis were as follows: miR-146b (AUC 0.94, 95% CI 0.87–1), miR-221 (AUC 0.79, 95% CI 0.68–0.9), miR-222 (AUC 0.76, 95% CI 0.63–0.89), and miR-15a (AUC 0.85, 95% CI 0.74–0.97). miR-146b showed 89% sensitivity (se) and 87% specificity (sp); miR-221 se = 68.4, sp = 90; miR-222 se = 73, sp = 70; and mi-R15a se = 72, sp = 80. MicroRNAs were associated with worst-prognosis clinicopathological characteristics in PTCs (*p* < 0.05), particularly for miR-222. Our data reveal a significant association between higher expression levels of miR-146b, miR-221, and miR-222 in the presence of the *BRAF* mutation (*p* < 0.001) and miR-146b (*p* = 0.016) and miR-221 (*p* = 0.010) with the *RAS* mutation, suggesting an interplay of these mutations with miRNAs expression. Despite this study having a relatively small sample size, overexpression of miRNAs in cytology may contribute to a more precise preoperative diagnosis. The miRNAs presented a good discriminative ability in PTC diagnosis. The association between the miRNAs expression profile and genetic alterations can be advantageous for an accurate diagnosis of DTCs/PTCs in FNAC.

## 1. Introduction

The incidence of DTCs, with over 95% of them diagnosed as papillary thyroid carcinoma (PTC) [1,2], has increased in recent years, related to the influence of environmental factors and the improvement of complementary diagnostic techniques, but without a change in mortality rate [3,4]. The prognosis of PTCs is usually favorable, but the association of some clinicopathological characteristics and the presence of some genetic mutations may affect their progression and behavior [5]. An accurate diagnosis in the pre-surgical phase is essential to optimize medical or surgical treatment and to avoid unnecessary surgeries and complications [6].

Ultrasound-guided fine needle aspiration cytology (US-FNAC) is the gold standard method for the diagnosis of nodular thyroid disease; however, up to 25% of nodules remain undiagnosed [7]. Cytological results in categories Bethesda II and VI present a very good performance to assess the benignity or malignancy of a thyroid nodule, respectively. The cytology diagnostic constraints are mainly significant in indeterminate Bethesda categories III (Atypia of Undeterminate Significance) and IV (Follicular Neoplasm). The Bethesda classification proposes a different risk of malignancy (ROM) for each category and different options for treatment and follow-up [8,9].

Both the European and American Thyroid Associations [10,11] ponder the inclusion of molecular tests in cytology by US-FNA. However, there is no consensus on the biomarkers panel to use in the pre-surgical diagnosis and prognosis of TC [12,13].

Another interesting area of knowledge in thyroid nodules is the evaluation of microRNAs (miRNAs) expression and its contribution to the diagnosis and prognosis of thyroid nodules [14,15,16]. They are stable molecules that can be detected in cytology and histology samples [17,18], in small amounts of fresh or stored material, and can also be studied in the blood [19,20].

MiRNAs are a class of numerous and small non-coding RNAs, with a length of approximately 22 nucleotides. MiRNAs are supposed to modulate 30% of the human genome and play an important role as global regulators of gene expression [21,22]. They interfere in posttranscriptional gene expression, by reducing the stability of the target messenger RNA (mRNA), and/or repressing its translation, and influencing intracellular regulatory processes. These events are involved in different stages of carcinogenesis, such as differentiation, proliferation, and apoptosis [23,24].

The knowledge of miRNA expression profiles in human tumors allowed us to identify specific “signatures” associated with diagnosis, prognosis, and response to treatment [25,26]. Unraveling their potential poses a substantial scientific challenge, underscoring the depth and importance of this tool.

A series of alterations in miRNAs biogenesis are revealed when a TC grows due to the action of oncogenes. Changes in the expression of miRNAs occur and determine the constitutive activation of the MAPK pathway [27]. OncomiRs (tumor promoters) and tumor suppressor miRNAs are distinct classes of miRNAs with contrasting roles in cancer biology. OncomiRs drive tumorigenesis by targeting tumor suppressor genes or activating oncogenic pathways, leading to uncontrolled cell proliferation, invasion, and metastasis. Conversely, tumor suppressor miRNAs counteract tumor development by targeting oncogenes or regulating key tumor-suppressive pathways, such as cell cycle arrest and apoptosis [21].

In our study, the first reference for the selection of the miRNAs was the study of Nikiforova (2008) [28] and an additional search on works related to our four selected miRNAs [28,29,30,31,32,33], in particular the work by Castagna (2019) [29], who selected the same miRs in fine-needle aspiration cytology.

Nikiforova et al. [28] showed the biological significance and interest of miRNAs analysis in normal and hyperplastic thyroid tissue, estimating that 32% of miRNAs are upregulated and 38% are downregulated in thyroid tumors. They compared it with thyroid normal tissue and identified specific upregulated and downregulated miRNAs implicated in PTC progress as miR-146b, miR-221, miR-222 (upregulated), and miR-15a (downregulated) [28]. The miRNA expression is tissue-specific, varying between different types of TC. Upregulated miR-146b, miR-221, and miR-222 are shown to downregulate proto-oncogene receptor tyrosine kinase (KIT), engaged in cell differentiation and growth, and in turn, miR-15a downregulates B-cell lymphoma 2 (BCL-2), controlling the proliferation and apoptosis of PTC through the AKT pathway [28].

Castagna, et al. [29] have shown, in cytologies of FNAs, that miR-146b, miR-221, and miR-222 are overexpressed in suspicious or malignant nodules, confirming the value of these miRNAs for the diagnosis of thyroid nodules. These miRNAs overexpressed were associated with tumor aggressiveness, extra thyroidal extension, recurrence, and metastases, constituting a specific signature of PTC [30]. The evaluation of miRNA expression may be helpful in the precocious diagnosis and in the evaluation of the biological behavior of PTC [31,32,33].

Although the identification of miRNAs expression profiles in thyroid cancer is being revealed, more studies are needed to integrate its diagnostic, prognostic, and predictive role in routine clinical practice. The identification of miRNAs and their target genes can allow us to envisage new and more personalized therapies [34,35].

This study aimed to analyze the expression of selected miRNAs (miR146b, miR221, miR-222, and miR15a) in thyroid nodules and their association with the presence of clinicopathologic features and genetic alterations (*TERTp*, *BRAF,* and *RAS* mutations). As an ultimate goal, we want to evaluate the reliability of molecular tests on US-FNAC as a pre-surgical diagnostic tool for the improvement of DTC diagnosis.

## 2. Materials and Methods

### 2.1. Study Design

This retrospective study was performed on a series of patients, submitted to surgery due to thyroid nodular disease suspicious of malignancy in a single non-oncologic hospital between 2013 and 2020. The criteria used for suspicion of malignancy were supported in clinical evaluation, image characteristics, and cytology Bethesda categories [7], in accordance with guidelines from ATA [10] and ETA [36]. Surgical protocols and recommendations for the extent of surgical treatment followed those guidelines [10,36]. 

The histological diagnosis obtained was composed of 13 histological benign lesions, included for comparative purposes (controls), and 93 malignant tumors (DTCs) (cases).

From those 106 patients, not all the specimens gave reproducible results in the molecular analysis, and we ended up with 98 cytology samples and 78 histology samples (formalin-fixed paraffin embedded (FFPE) tissues), which were paired cyto-histology samples in 70 cases (13 paired benign lesions and 57 DTCs), as shown in Supplementary Table S1.

Study inclusion criteria: patients diagnosed with benign thyroid nodules (controls) or differentiated thyroid carcinoma (cases), after surgery, over 18 years old, of both genders, and with available histological and cytological samples.

Study exclusion criteria: patients diagnosed with dysfunction goiter, smaller than 1 cm papillary carcinoma, and patients diagnosed with other malignant neoplasms at the time of thyroid surgery.

The epidemiologic and clinicopathological characteristics of patients and tumors were gathered from the clinical and histopathological reports from the reference hospital.

Two independent pathologists reviewed the cytology and histology samples, following the 4th edition of the World Health Organization (WHO) Classification of Tumors of Endocrine Organs [37]. 

### 2.2. MicroRNAs Expression Profile Analysis

#### 2.2.1. MicroRNA Extraction from FFPE Tissues and FNAC Samples

The initial step is different for FFPE tissues and FNAC samples. For FFPE tissues, a slide (10 μm cut) was deparaffinized, and the tumor area was manually micro-dissected. For FNAC samples, the entire smears were manually scraped. For miRNAs extraction, the miRNeasy Mini kit (1038703 Qiagen, Venlo, The Netherlands) was applied in accordance with the manufacturer’s instructions. 

To determine the RNA concentration the NanoDrop (ND-1000, Thermo Fisher Scientific, Vilnius, Lithuania) spectrophotometer was used. RNA handling was performed on ice.

#### 2.2.2. Reverse Transcription and Quantitative Real Time PCR (RT-qPCR)

To achieve complementary deoxyribonucleic acid (cDNA) synthesis, the TaqMan^®^ Advanced miRNA cDNA Synthesis Kit (refª A28007, Applied Biosystems, Willmington, DE, USA) was used in accordance with the manufacturer’s instructions. 

RT-qPCR was used to determine the relative expression levels of miR-146b, miR-221, miR-222, and miR-15a (refª A25576, ThermoScientific, Waltham, MA, USA) [38]. MiR-16 (refª A25576, ThermoScientific, Waltham, MA, USA) was performed as an endogenous control for normalization of the miRNAs expression values [39]. The quantification of gene expression was determined by the 2^−ΔΔCT^ method [40]. 

RT-qPCR was performed using the TaqMan^®^ Fast Advanced system (refª 4444557, ThermoScientific, Waltham, MA, USA) according to the manufacturer’s instructions on a QuantStudio 5 Real-Time PCR System (ThermoScientific, Waltham, MA, USA). All samples were duplicated.

### 2.3. Genetic Analysis

#### 2.3.1. DNA Extraction

Genomic DNA was extracted using the GRS Genomic DNA BroadRange Kit (refª GK06.0100 GRiSP Research Solutions, Porto, Portugal) or the QIAmp^®^ DNA Investigator Kit (refª 56504 Qiagen, Venlo, The Netherlands) for FFPE and US-FNACs, respectively, following the manufacturer’s instructions. The quantification of isolated DNA was performed with the NanoDropTM One UV185 Vis Spectrophotometer (Thermo Fisher Scientific Inc.).

#### 2.3.2. Mutational Screening

For genetic characterization of the lesions, PCR (SimpliAmp^TM^ Thermalcycler, Applied Biosystems, Willmington, DE, USA) and Sanger sequencing (Applied Biosystems 3130/3130XL Genetic Analyzer, Foster City, CA, USA) were used. Mutations in *TERTp*, *BRAF*, and *RAS* (*NRAS*, *HRAS*, and *KRAS*) were evaluated by PCR/sequencing with primers for the most frequent regions mutated in thyroid carcinoma, as previously described [41,42]. A new independent analysis validated all detected mutations.

### 2.4. Clinicopathological Characteristics

Only PTCs were considered in the statistical analysis of the miRNA expression and clinicopathological characteristics of the nodules. The tumor’s clinicopathological features analyzed were: tumor size, extra thyroidal extension, capsular invasion, vascular and lymphatic invasion, inflammatory infiltrate, fibrosis, oncocytic component, psammoma bodies, tall cell component, calcification, necrosis, focality, and lymph node metastases. 

### 2.5. Statistical Analysis

An exploratory analysis was performed with categorical variables presented with frequencies (percentages) and quantitative variables with mean, standard deviation (SD) or median, and percentiles (25–75) and/or range (minimum–maximum), as appropriate. The Student’s *t*-test and non-parametric Mann–Whitney test were performed to compare groups regarding quantitative variables, as appropriate. The Chi-square or Fisher’s exact tests were used in the case of categorical variables, as required.

The discriminative ability of biomarkers regarding malignancy was estimated using sensitivity, specificity, and positive and negative predictive values with 95% confidence intervals. The level of significance *p* < 0.05 was considered. SPSS software version 27.0 (IBM Corp. was released 2020. IBM SPSS Statistics for Windows, Version 27.0., Armonk, NY, USA: IBM Corp.) was used to perform data analysis. 

## 3. Results

### 3.1. Series Description 

#### 3.1.1. Epidemiologic Data

From the 106 patients, 89 (84%) were female, with a mean age at diagnosis of 52.2 (16.4) years (range 18–84), and 17 (16%) were male, with a mean age of 55.2 (13.5) years (range 35–79). The distribution of age was the same across categories of gender (*p* = 0.564) and across categories of histological diagnosis (*p* = 0.503).

The tumor size presented a mean value of 33.1 mm (11.94) for benign lesions and 28.3 mm (14.76) for malignant lesions; the distribution of tumor size was the same across categories of histological diagnosis (*p* = 0.152).

Out of 106 operated patients, final histology diagnoses were benign in 13 (12.3%) cases and malignant lesions (DTC) in 93 (87.7%) cases. PTCs were present in 81 (87.1%) cases, being the larger group in malignant histology.

In accordance with the Bethesda classification, cytology samples were distributed, respectively: I. Non-Diagnostic (ND), 3 samples (2.8%); II. Benign (B), 23 samples (21.7%); III. AUS, 23 samples (21.7%); IV. FN, 29 samples (27.4%); V. Suspicious for Malignancy (SM), 14 samples (13.2%); and VI. Malignant (M), 14 samples (13.2%). 

Fifty-two (49.1%) nodules whose cytological result was indeterminate (Bethesda III and IV) corresponded in histology to one (1.9%) benign and to 51 malignant lesions (98.1%).

The cytology samples distribution within histological subtypes in all series is presented in Table 1.

#### 3.1.2. MiRNAs Profile in Cytology Samples

The miRNAs expression levels of the four miRNAs (miR-146b, miR-221, miR-222, and miR-15a) in cytology samples by Bethesda categories are summarized in Supplementary Table S2. The levels of miRNAs expression observed across Bethesda categories exhibit elevated values, characterized by high median values, progressing from category I to category VI. Expressive variations in maximum values were noted among all miRNAs for each category, with the exception of miR-15a, which precludes statistically significant differences.

Indeterminate nodules (Bethesda III and IV) and suspicious or malignant nodules (Bethesda V and VI) with higher miRNA expression levels proved to be malignant in histology, except in one benign case (Bethesda IV), as seen also in Table 1.

**Figure 1:**
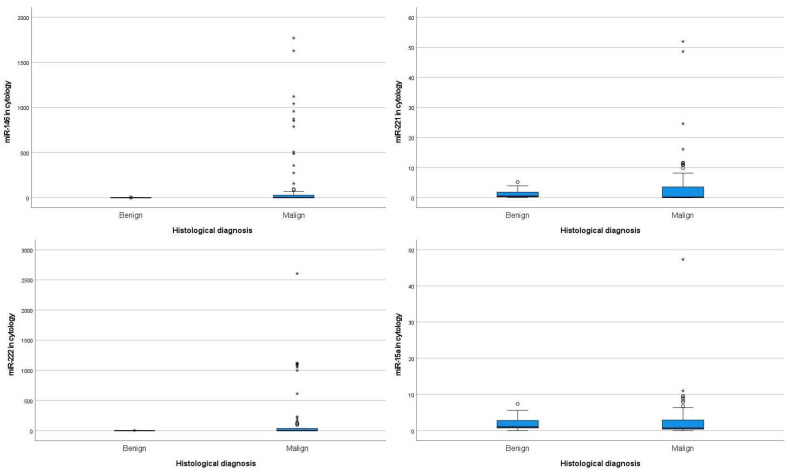
miRNAs profile in cytology samples by histological final diagnosis in all series. *—Severe outlier values of miRNAs, circle—moderate outlier values of miRNAs.

The miRNA expression levels of the four miRNAs in cytology samples by histology diagnosis, in all series, are summarized in Figure 1 and Supplementary Table S3. The miRNAs profile observed in cytology samples showed a tendency to be overexpressed in malignant tumors when compared with benign lesions, with a higher median for miRNAs 146b and 222 and wider amplitude of expression for all miRNAs in malignant tumors, although without statistical significance.

When we analyzed miRNA values obtained in cytology samples in accordance with the final diagnosis of malignancy, we noted that the malignant cases presented miRNA expression values above those obtained in benign cases. The number of malignant cases that presented (in the cytology sample) values of miRNAs expression above the maximum levels obtained for benign tumors were 34.1% (*n* = 29) cases for miRNA-146b, 17.4% (*n* = 16) for miRNA-221, 35.4% (*n* = 29) for miRNA-222, and 10.3% (*n* = 9) for miRNA-15a, as shown in Table 2.

Malignant tumors presented, in cytology, higher values of miRNA expression when compared with benign tumors. The five highest extreme values for miRNAs expression in cytology by histological diagnosis are shown in Supplementary Table S4.

#### 3.1.3. MiRNAs Profile in Histology Samples

**Figure 2:**
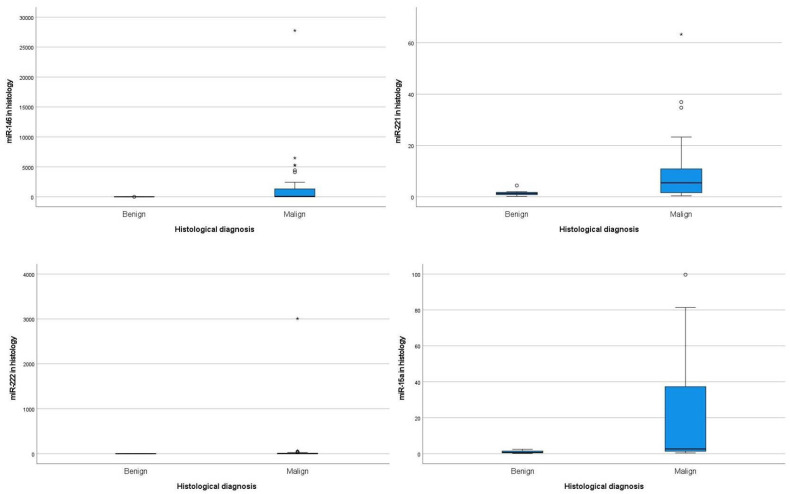
miRNAs expression levels in histology samples in all series. *—Severe outlier values of miRNAs, circle—moderate outlier values of miRNAs.

The miRNAs expression levels of miR-146b, miR-221, miR-222, and miR-15a in histology samples in all series are shown in Figure 2 and Table 3.

The miRNAs profile observed in histologies presented a statistically significant difference between benign and malignant tumors for the four miRNAs (Table 3), showing higher median values and a wider amplitude of expression in malignant tumors.

The miRNAs expression levels of the four miRNAs in histology samples within histological subtypes are summarized in Supplementary Table S5.

Most histological subtypes of DTCs were PTCs (81/93) and presented higher median values and a wider amplitude of expression for all miRNAs. Twelve cases were non-PTCs, of which two cases of HCC presented higher median values and a wider amplitude of expression for miRNAs 221 and 222.

### 3.2. MiRNA Expression and Mutations in PTCs

For the analysis of the association between miRNAs expression levels and the presence of genetic mutations in cytology and histology samples, only PTCs were considered because of the small number of other subtypes of DTC.

In cytology samples, the miRNAs profile revealed a higher median expression value in the presence of genetic mutations, as shown by miRNA-146b and the presence of *TERTp* and *BRAF* mutations, miRNA-221 and miRNA-222 associated with *BRAF* mutations, and miRNA-15a with *TERTp* and *RAS* mutations, although no statistically significant association was observed (Supplementary Table S6).

In histology samples, miRNAs expression revealed a higher median value in the presence of almost all genetic mutations, and statistically significant associations between miRNAs expression levels and the presence of genetic mutations were obtained. MiRNA-146 and miRNA-221 expression levels were significantly associated with the presence of *BRAF* and *RAS* mutations, miRNA-222 with the presence of *BRAF* mutations, and miRNA-15a with the presence of *TERTp* and *RAS* mutations (Table 4).

### 3.3. MiRNA Expression and Clinicopatological Features in PTCs

The association between microRNAs expression and clinicopathological features of the tumors was evaluated in PTCs, with the results shown in Supplementary Table S7A–D.

No statistically significant differences were obtained for gender or age, and the distribution of tumor size was the same across microRNAs expression at diagnosis.

MiR-146b expression showed an association with capsular invasion, psammoma bodies (*p* = 0.019), and calcification (*p* = 0.010). Overexpressed miR-221 was associated with capsule invasion and vascular invasion (*p* < 0.001), the oncocytic component (*p* = 0.002), and calcification (*p* = 0.031). miR-222 expression presented associations with the presence of extra thyroidal extension, capsule invasion, vascular invasion, lymphatic invasion, oncocytic component, psammoma bodies, inflammatory infiltrate, tall cell component, calcification, and focality (*p* < 0.05 for all parameters). miR-15a expression presented a significant association with capsular invasion and focality (*p* = 0.41).

### 3.4. The Discriminative Ability of miRNAs in Histology for Malignancy

**Figure 3:**
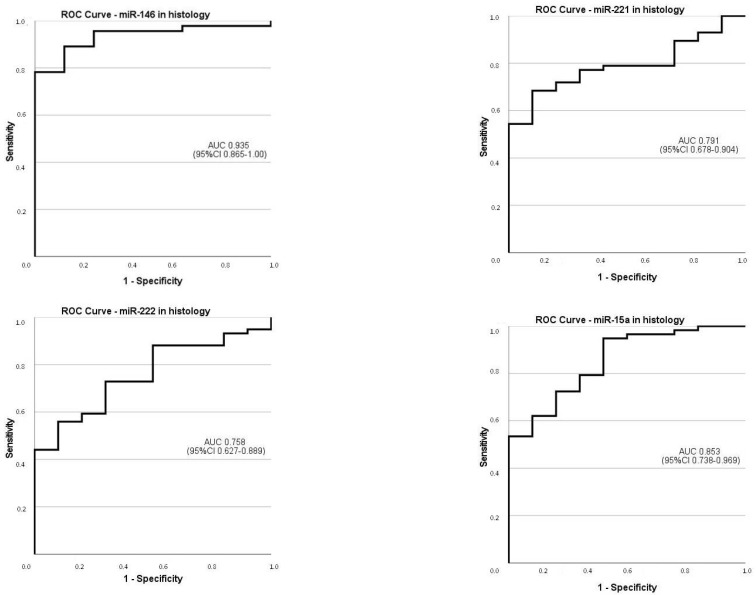
The discriminative ability of miRNAs in histology for the diagnosis of malignancy in papillary thyroid carcinomas.

The discriminative ability of the miRNAs profile for the diagnosis of malignancy in our series was analyzed only for PTCs because of the small number of other malignant subtypes of DTC, as shown in Table 5 and Figure 3.

Area under the curve (AUC) for all miRNAs showed acceptable to exceptional discriminative ability regarding histological diagnosis, with estimates ranging from 75.8% (95% CI: 62.7–88.9) for miR-222 to 93.5% (95% CI: 86.5–100) for miR-146b.

Regarding PTCs diagnosis and considering the cutoff values obtained by maximizing sensitivity and specificity, all miRNAs exhibited sensitivity (se) and specificity (sp) over 70%, with higher estimates for miR-146b (89.1% se and 87.5% sp). The positive predictive value (PPV) was over 93.5% for all miRNAs and attained a maximum of 97.6% for miR-146b. The negative predictive value (NPV) was over 30% for all the miRNAs, with 58.3% for miR-146b.

The statistical analysis of combined miRNAs did not improve the discriminative ability of miRNAs for PTC diagnosis in our study. The analysis of miRNAs expression in 70 paired cyto-histology samples did not show statistical significance.

## 4. Discussion

The expression of miRNAs is deregulated in many types of human cancer, including thyroid cancer, leading to alterations in different cellular processes, such as cell differentiation, proliferation, migration, and invasion [26]. Together with genetic alterations, miRs have been considered as new biomarkers in TC and are regarded as possible therapeutic targets in the same cases of malignancy [42,43,44].

In 2022, the 5th WHO classification of thyroid neoplasms [45] and in 2023, the 3rd Bethesda Classification [8], included genetic and molecular biomarkers for pre-surgical diagnosis to refine the diagnostic categories and their risk of malignancy. However, in addition to the lack of consensus on which panels of molecular biomarkers to use, their implementation in clinical practice is not yet possible in the vast majority of countries worldwide for technical and economic reasons.

The aim of this study was to evaluate the expression levels of four miRs in cytology via US-FNAC and in histology samples of patients with thyroid nodular disease that underwent surgery, based on clinical evaluation, imageology features, and cytological criteria for suspicion of malignancy. We selected three miRNAs previously described as upregulated in TC (miR-146b, miR-221, and miR-222) and one categorized as downregulated (miR-15a) for expression profile analyses [46].

We observed a broad spectrum of variation in miRNA expression levels, evident both in cytology and in histology analyses. In histology, this study reveals a significant overexpression of miRNAs in malignant tumors when compared with benign lesions. In TC, overexpression of these specific miRNAs has already been described in the literature in association with thyroid cell transformation [30,47].

In cytology samples, miRNAs showed a consistent trend for overexpression in malignant tumors compared with benign lesions, displaying higher median values across Bethesda categories I to VI. Notably, there were considerable differences in maximum values within each Bethesda category for all miRNAs, except miR-15a, despite no statistical significance has been achieved. In histology samples, all four miRNAs were overexpressed, with a statistical significance between malignant tumors and benign lesions. The microRNA expression profile in cytology and in histology presented different levels with high amplitudes, which may reflect tumor multifocality, heterogeneity, and/or low cellular representativity, particularly marked on cytology samples, with a lower percentage of cells (neoplastic) causing an increase in false negative results.

When the values of miRNAs expression in cytology for malignant tumors under maximum levels for benign lesions (Table 2) were considered, the cytological information taken together with clinical evaluation and imageology could improve the diagnosis in one-third of cases for miRNAs 146b and 222, almost 20% for miR-221, and even 10% for miR-15a. Those alterations in miRNAs may contribute to a precocious diagnosis of malignancy in thyroid nodules, a reduction in the number of FNACs, and better surgical options.

Indeterminate nodules are the reason why we need better diagnostic tools in FNAC. However in our series, the indeterminate nodules presented a high number of malignant cases (due to selection bias), representing a major limitation as mentioned ahead, because case selection was done based in a histology diagnosis of malignancy (or benignity in the control cases), and these patients underwent surgery due to clinical reasons besides FNACs results (e.g., US features). In indeterminate nodules, miRNAs showed a tendency for overexpression in cytology and with statistical significance in histology, reflecting their malignity in our series.

We found a tendency for upregulation in miR-146b in cytology and statistically significant in histology, which is in line with other studies [48,49,50]. MiR-146b is described as targeting SMADA and IRAK1, causing downregulation and increasing cell proliferation and migration in PTCs [51].

The expression levels of miR-221 and miR-222 in cytology were higher in malignant tumors than in benign lesions and reached statistical significance in histology, in accordance with Visone R. et al. [52]. Both miR-221 and miR-222 target CDKN1B (p27Kip1 protein), which is a significant regulator of the cell cycle, and KIT, which is implicated in cell differentiation and growth. The overexpression of miR-221 and miR-222 triggered the papillary thyroid carcinoma cell line (TPC-1) to progress into the S phase of the cell cycle, causing a downregulation of the p27Kip1 protein. However, the inhibition of miR-221 and miR-222 expression increases p27Kip1 protein levels. These results suggest the involvement of these miRs in the control of the cell cycle, regulating p27Kip1 protein levels [52].

MiR-15a has been considered downregulated in thyroid cancer. Nevertheless, in our study, we observed an upregulation, suggesting that miR-15a has a gain function in PTCs. Jin J et al. [25] showed an association between miR-15a low expression levels and PTCs, and the possible effect of these miRNAs in the promotion of several stages of TC development (proliferation, invasion, and survival). On the contrary, Lu Z et al. [34] showed that the upregulation of miR15a suppresses BCL-2 expression. In an in vivo study, when miR15a was upregulated, it inhibited cell proliferation and invasion, promoting apoptosis. They concluded that the overexpression of miR-15a inhibited tumor progression by regulating the AKT pathway. These discrepant results indicate the need for further studies to establish the role of miR-15a in thyroid cancer.

Several statistically significant associations between clinicopathological and molecular characteristics of the PTCs were already described for *BRAF* mutations [53] and *TERTp* mutations [54], both associated with features of worse prognosis, whereas *RAS* mutations are associated with benign or low-risk tumors [55,56]. Our data proved a significant association between the expression levels of miR-146b, miR-221, and miR-222 and the presence of *BRAF* mutations in PTCs. Our findings are in accordance with Yang S. I. et al. [57], who showed that the expression of miR-146b was significantly associated with the presence of *BRAF* mutations. Sun Y. et al. [58] revealed that miR-146b, miR-221, and miR-222, together with miR-181, were upregulated in PTCs patients with *BRAF* mutations. These studies suggest that the concomitant presence of *BRAF* mutations with alterations in miRNAs expression may result in a connection or influence on the development of TC. Our results showed a significant association between the expression levels of miR-146b, miR-221, and miR15a, and the presence of *RAS* mutations in PTCs. The presence of *RAS* mutations confirms a neoplasm but does not differentiate between benign, malignant, and low-risk neoplasms, whose analysis was beyond the scope of our study, as carried out by others [59]. Curiously, only miR-15a was significantly associated with the presence of *TERTp* mutations in PTCs, suggesting that *TERTp*-mutated PTCs can have a different repertoire of microRNA expression.

The (over)expression of the four microRNAs was significantly associated with several clinicopathological features in PTCs, namely capsular invasion and vascular and lymphatic invasion, which were related to aggressive tumor behavior in accordance with others [29,33]. For example, upregulation of miR-146b was previously associated with tumor aggressiveness and poor clinicopathological characteristics, such as extra thyroidal extension and capsule invasion, the existence of lymph nodes, or distant metastasis [60].Our miRNA expression analysis presents some limitations, namely the retrospective nature of the study and the small size of the series, both in cytology (lower representative neoplastic cells) and in histology, due to the small number of non-PTC subtypes. Both mentioned limitations, plus the great variability of miR expression levels in cytology and histology, did not allow the comparison in paired cyto-histological cases. Another limitation was the bias in the selection of indeterminate nodules in our series, making their study unfeasible, as conducted by others [15,18,47]. However, our results suggest that cytologies with overexpression levels of miRs have a higher risk of being malignant lesions, and miRNA evaluation may contribute to a pre-surgical diagnosis, changing the clinical and therapeutic decision.

Our results of the miRNA expression profile in thyroid nodules may enhance the potential clinical utility of molecular testing in FNACs, anticipating a diagnosis of malignancy and predicting tumor behavior. By integrating molecular information, clinicians can make better informed decisions and optimize treatment strategies.

## 5. Conclusions

Our results confirm a deregulation of the expression levels of selected miRNAs in TC through their potential influence on their target genes, causing cell differentiation, proliferation, and survival. Despite the miRNAs profile’s limited ability to exclude malignancy, we may suspect malignancy in cytology samples with high expression levels of miRs, contributing to reducing repeated US-FNAC and optimizing the pre-surgical diagnosis. Moreover, the associations identified between miRNAs expression and clinicopathological features, on the one hand, and the genetic profile of PTCs, on the other hand, suggest that molecular analysis in cytology via FNA can contribute to a better understanding of tumor genetic profile and behavior.

Current results suggest that miRNAs profile analysis in US-FNAC can improve a precocious and accurate diagnosis of DTCs/PTCs and, therefore, is likely to contribute to an effective treatment of TC. Yet, confirmation is pending from the analysis of a larger series.

## Figures and Tables

**Table 1:** Cytology samples distribution within histological subtypes in all series.

Cytology Diagnosis *n* = 106	Histology Diagnosis *n* = 106						
	Benign	WDT-UMP	NIFT	PTC	FTC	HCC	Total
1. ND	0 (0%)	0 (0%)	1 (0.9%)	2 (1.9%)	0 (0%)	0 (0%)	3 (2.8%)
2. Benign	12 (11.3%)	0 (0%)	2 (1.9%)	7 (6.6%)	1 (0.9%)	1(0.9%)	23 (21.7%)
3. AUS	00 (0%)	0 (0%)	0 (0%)	23 (21.7%)	0 (0%)	0 (0%)	23 (21.7%)
4. FN	01 (0.9%)	2 (1.9%)	1 (0.9%)	23 (21.7%)	1 (0.9%)	1 (0.9%)	29 (27.4%)
5. SM	00 (0%)	0 (0%)	0 (0%)	12 (11.3%)	2 (1.9%)	0 (0%)	14 (13.2%)
6. Malignant	00 (0%)	0 (0%)	0 (0%)	14 (13.2%)	0 (0%)	0 (0%)	14 (13.2%)
Total	13 (12.3%)	2 (1.9%)	4 (3.8%)	81 (76.4%)	4 (3.8%)	2 (1.9%)	106 (100%)

Legend: WDT-UMP: Well-differentiated thyroid tumor of uncertain malignant potential; NIFT: noninvasive follicular thyroid neoplasm with papillary-like nuclear features; PTC: papillary thyroid carcinoma; FTC: follicular thyroid carcinoma; HCC: Hürthle cell carcinoma; ND: Non-diagnostic; AUS: Atypia of Undetermined Significance; FN: Follicular Neoplasm; SM: Suspicious for malignancy; and M: Malignant.

**Table 2:** Frequencies of miRNAs expression in cytology by histology diagnosis in all series.

miRNAs in Cytology	Frequencies of miRNAs Expression by Histology Diagnosis				
	Final Diagnosis	Median *	Under Maximum value * *n* = (%)	Over Maximum Value * *n* = (%)	Total *n* = (%)
miRNA146			≤4.394	>4.394	96 (100)
	Benign	0.308	11 (100)	-	11 (11.5)
	Malignant	0.489	55 (64.7)	30 (35.3)	85 (88.5)
miRNA221			≤5.242	>5.242	97 (100)
	Benign	0.535	11 (100)	-	11 (11.3)
	Malignant	0.172	70 (81.4)	16 (18.6)	86 (88.7)
miRNA222			≤6.358	>6.358	97 (100)
	Benign	0.914	11 (100)	-	11 (11.3)
	Malignant	1.46	56 (65.1)	30 (34.9)	86 (88.7)
miRNA15a			≤7.39	>7.39	98 (100)
	Benign	1.053	11 (100)	-	11 (11.2)
	Malignant	0.686	77 (88.5)	10 (11.5)	87 (88.8)

* The 2^−ΔΔCT^ method quantification of miRNA expression normalized to miR-16.

**Table 3:** miRNAs expression in histology samples by histology diagnosis in all series.

miRNAs in Histology			Histology Diagnosis			
	*n*	Final Diagnosis	Median *	P25–P75 *	Min–Max Value *	*p*-Value
miRNA146	60					0.002
	8	Benign	0.66	0.392–2.455	0.322–5.341	
	52	Malignant	44.529	3.215–907.373	0.016–27,755	
miRNA221	76					0.008
	10	Benign	1.312	0.781–1.798	0.192–4.395	
	66	Malignant	4.297	1.475–9.695	0.012–63.304	
miRNA222	78					0.017
	10	Benign	1.067	0.645–2.544	0.328–4.347	
	68	Malignant	3.409	1.075–13.027	0.114–3006.772	
miRNA15a	77					0.002
	10	Benign	0.683	0.510–1.619	0.282–2.513	
	67	Malignant	2.111	1.204–37.237	0.230–99.640	

* The 2^−ΔΔCT^ method (quantification of miRNA expression normalized to miR-16). Legend: P25–P75: percentile 25 and percentile 75. Min–max: minimum and maximum values.

**Table 4:** Association between miRNAs expression levels and genetic mutations in PTCs.

miRNAs in Histology								
Genetic Mutations in Histology	miRNA-146		miRNA-221		miRNA-222		miRNA15a	
	*n*	Median *	*n*	Median *	*n*	Median *	*n*	Median *
*TERTp*	54		67		69		68	
Absent	48	29.856	59	2.99	61	3.076	60	1.981
Present	6	60.203	8	6.31	8	3.958	8	37.494
*p*-value		0.563		0.451		0.708		**0.033**
*BRAF*	54		67		69		68	
Absent	39	9.9	49	1.82	50	1.548	50	2.087
Present	15	133.574	18	8.625	19	14.006	18	2.257
*p*-value		**0.02**		**0.001**		<0.001		0.792
*RAS*	54		67		69		68	
Absent	41	26.511	53	2.464	55	2.325	54	1.713
Present	13	716.144	14	10.9	14	5.284	14	24.767
*p*-value		**0.016**		**0.01**		0.144		**0.026**

* The 2^−ΔΔCT^ method (quantification of miRNA expression normalized to miR-16). *p*-values in bold to highlight statistical significance. Legend: *TERTp*: telomerase reverse transcriptase promoter; *BRAF*: B-Raf proto-oncogene, serine/threonine kinase; *NRAS*: *NRAS* proto-oncogene, GTPase; *HRAS*: *HRAS* proto-oncogene, GTPase; and *KRAS*—*KRAS* proto-oncogene, GTPase.

**Table 5:** The discriminative ability of miRNAs for PTC diagnosis.

miRNAs in Histology	Papillary Thyroid Carcinomas					
(*n*)	Cutoff	AUC (95% CI)	Se % (95% CI)	Sp % (95% CI)	PPV % (95% CI)	NPV % (95% CI)
miRNA-146b	3. 070	93.5 (86.5–100)	89.1 (76.4–96.3)	87.5 (84–99.2)	97.6 (84–99.2)	58.3 (35.6–98.4)
miRNA-221	1.762	79.1 (67.8–90.4)	71.9 (58.5–83)	80 (44.4–97.5)	95.3 (80.4–97.5)	33.3 (21.5–82.9)
miRNA-222	1.392	75.8 (62.7–88.9	72.9 (59.7–83.6)	70 (34.8–93.3)	93.5 (76.6–96.5)	30.4 (19.5–72.4)
miRNA-15a	1.537	85.3 (73.8–96.9)	72.4 (59.1–83.3)	80 (44.4–97.5)	95.5 (80.7–97.6)	33.3 (21.6–82.9)

AUC: area under the curve; Se: sensibility; Sp: specificity; PPV: predictive positive value; and NPV: negative predictive value.

## Data Availability

Data are contained within the article and Supplementary Materials.

## Supplementary Materials

The following supporting information can be downloaded at: https://www.mdpi.com/article/10.3390/genes15030389/s1, Supplementary Table S1. Series composition for miRNA analysis; Table S2. MiRNAs expression in cytology samples by Bethesda categories in all series. Table S3. MiRNAs expression in cytology samples by histology diagnosis in all series. Table S4. Five highest extreme frequencies of miRNAs expression in cytology samples by histology diagnosis in all series. Table S5. MiRNAs expression in histology within histological subtypes in all series. Table S6. Association of the miRNAs expression levels and the presence of genetic mutations in cytology samples in PTCs. Table S7. A. Associations between miR-146b expression and clinicopathological features in Papillary Thyroid Carcinomas. Supplementary Table S7. B. Associations between miR-221 expression and clinicopathological features in Papillary Thyroid Carcinomas. Supplementary Table S7. C. Associations between miR-222 expression and clinicopathological features in Papillary Thyroid Carcinomas. Supplementary Table S7. D. Associations between miR-15a expression and clinicopathological features in Papillary Thyroid Carcinomas.
