# Supplementary material for: Cyto-Histological Profile of MicroRNAs as Diagnostic Biomarkers in Differentiated Thyroid Carcinomas
Source: Genes (Basel). 2024 Mar 21;15(3):389. doi: 10.3390/genes15030389 (PMC10970297; doi:10.3390/genes15030389)
Supplement: Supplementary file 1 [file genes-15-00389-s001.zip › Supplementary Tables MS2 miRNAs 200324.pdf]

Supplementary Table S1. Series composition for miRNA analysis.

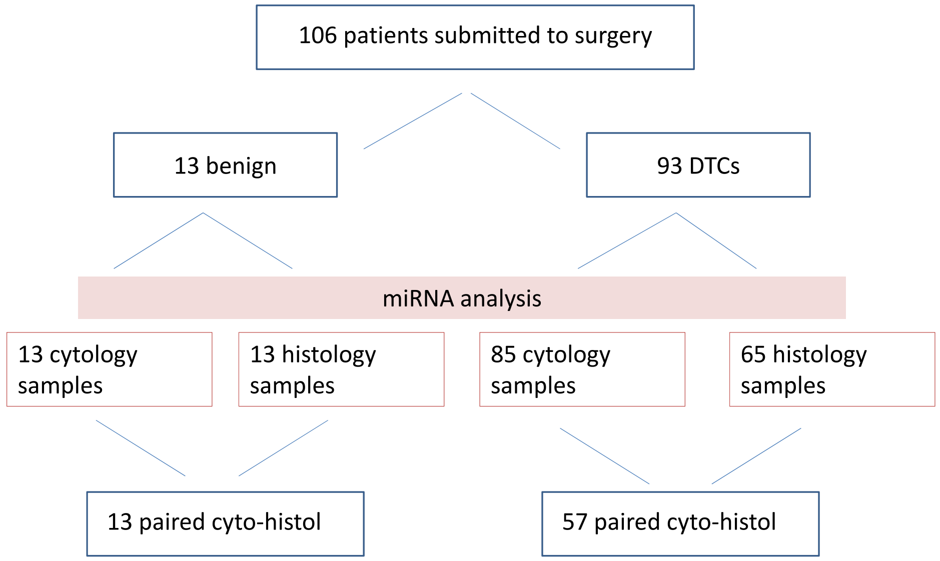

Supplementary Table 1. Series composition for miRNA analysis

Supplementary Table S2. MiRNAs expression in cytology samples by Bethesda categories in all series.

miRNAs in cytology	By Bethesda categories				
	n	Cytology	Median*	P25 - P75*	Min - Max value*
miRNA146	96				
	03	ND	0.005	0.000 - 0.000	0.000 –0.21
	20	Benign	0.161	0.071 – 1.339	0.000 – 853.8
	47	AUS + FN	0.756	0.079 – 7.395	0.000 – 1768,34
	26	SM+ M	1.275	0.004 – 138.81	0.000 – 1627.93
miRNA221	97				
	03	ND	0.062	0.000 – 0.000	0.000 –0.081
	20	Benign	0.237	0.062 – 1.802	0.000 – 11.033
	48	AUS + FN	0.169	0.043 – 1.095	0.000 – 51.976
	26	SM+ M	0.400	0.000 – 8.265	0.000 – 24.626
miRNA222	91				
	03	ND	0.903	0.000 –0.000	0.000 –1.501
	18	Benign	1.951	0.830 –5.545	0.000 – 1120.686
	44	AUS + FN	1.045	0.343 – 14.592	0.000 – 2604.770
	26	SM+ M	7.581	.004 – 121.022	0.000 – 1111.663
miRNA15a	98				
	03	ND	0.412	0.000 –0.000	0.000 – 0.772
	21	Benign	0.745	0.408 – 1.148	0.000 – 7.390
	48	AUS + FN	1.198	0.475 – 4.772	0.000 – 47.341
	26	SM+ M	0.691	0.000 – 2.629	0.000 – 7.831

* The 2-ΔΔCT method was used to quantification of miRNA expression normalized to miR-16.

Legend: ND: Non-diagnostic, AUS: Atypia of Undetermined Significance, FN: Follicular Neoplasm, SM: Suspicious for malignancy and M: Malignant. P25-75: percentile 25 and percentile 75. Max-Min: maximum and minimum value.

MiRNAs expression in cytology	Histology diagnosis					
	n	Final Diagnosis	Median*	P25 - P75*	min - max value*	p-value
miRNA146	96					0.895
	11	Benign	0.308	0.141 - 1.530	0.086 - 4.394	
	85	Malignant	0.489	0.045 - 16.80	0.000 - 1768.34	
miRNA221	97					0.169
	11	Benign	0.535	0.155 - 2.351	0.035 - 5.242	
	86	Malignant	0.172	0.039 - 2.636	0.000 - 51.976	
miRNA222	97					0.402
	11	Benign	0.914	0.506 - 2.493	0.089 - 6.358	
	86	Malignant	1.460	0.330 - 32.199	0.000 - 2604,77	
miRNA15a	98					0.402
	11	Benign	1.053	0.745 – 4.349	0.016 - 7.390	

Supplementary Table S3. MiRNAs expression in cytology samples by histology diagnosis in all series.

	87	Malignant	0.686	0.415 – 2.999	0.000 - 47.341	

* The 2-ΔΔCT method was used to quantification of miRNA expression normalized to miR-16.

Legend: P25-75: percentile 25 and percentile 75. Max-min: maximum and minimum value

Supplementary Table S4. Five highest extreme frequencies of miRNAs expression in cytology samples by histology diagnosis in all series.

Histology diagnosis	Highest values of miRNAs expression* in cytology			
	miRNA-146	miRNA-221	miRNA-222	miRNA-15a
Benign	4.394	5.242	6.358	7.390
	2.881	3.978	3.139	5.620
	1.529	2.351	1.846	4.349
	0.770	1.401	1.384	1.242
	0.610	0.677	0.914	1.193
Malignant	1768.341	51.976	2604.770	47.341
	1627.930	48.630	1120.686	11.342
	1121.931	24.626	1112.533	10.989
	1041.914	16.130	1111.663	9.556
	958.423	11.653	1106.747	9.486

* The 2-ΔΔCT method was used to quantification of miRNA expression normalized to miR-16.

Supplementary Table S5. MiRNAs expression in histology within histological subtypes in all series.

miRNAs in histology* n=106	Histological subtypes					
	Benign n=13	WDT-UMP n=2	NIFT n=4	PTC n=81	FTC n=4	HCC n=2
miRNA-146						
n= 60	n=8	n=1	n=2	n=46	n=2	n=1
Median*	0.66	-	0.226	65.667	0.227 – 0.	-
P25-P75*	0.392 – 2.456	-	0.142 – 0.	8.351 – 1316.098	0.025 – 0.	-
Min – Max *	0.322 – 5.341	-	0.142 – 0.310	0.016 –27755.752	0.025 – 0.428	-
miRNA-221						
n= 76	n=10	n=1	n=4	n=57	n=2	n=2
Median*	1.312	-	1.918	5.45	0.115	6.278
P25-P75*	0.781 – 1.798	-	0.385 – 4.092	1.582 –10.900	0.108 – 0.	3.297 – 0.
Min – Max*	0.192 – 4.395	-	0.012 – 4.678	0.368 – 63.304	0.108 – 0.124	3.297 – 9.260
miRNA-222						
n= 78	n=10	n=1	n=4	n=59	n=2	n=2
Median*	1.067	-	2.465	3.511	0.134	16.21
P25-P75*	0.645 – 2.544	-	0.626 – 9.693	1.183 – 13.315	0.124 – 0.	7.7 – 0.
Min – Max*	0.328 – 4.348	-	0.271 – 11.843	0.115 – 3006.7	0.124 – 0.143	7.7 – 0. 24.72
miRNA-15a						
n= 77	n=10	n=1	n=4	n=58	n=2	n=2
Median*	0.683	-	1.020	2.682	1.594	0.236
P25-P75*	0.510 – 1.610	-	0.835 – 2.177	1.396 – 37.388	1.439 – 0.	0.231 – 0.
Min – Max*	0.282 – 2.514	-	0.779 – 2.556	0.479 – 99.641	1.439 – 1.747	0.231 – 0.241

* The 2-ΔΔCT method was used to quantification of miRNA expression normalized to miR-16.

Legend: WDT-UMP: Well differentiated thyroid tumor of uncertain malignant potential; NIFT: noninvasive follicular thyroid neoplasm with papillary-like nuclear features; PTC: papillary thyroid carcinoma; FTC: follicular thyroid carcinoma; HCC: Hürthle cell carcinoma: not available data to estimate summary measures. P25-75: percentile 25 and percentile 75. Max-min: maximum and minimum value

Supplementary Table S6. Association of the miRNAs expression levels and the presence of genetic mutations in cytology samples in PTCs.

Genetic mutations in Cytology	miRNAs in Cytology							
	miRNA-146		miRNA-221		miRNA-222		miRNA-15a	
	n	Median*	n	Median*	n	Median*	n	Median*
TERTp	84		85		80		86	
Absent	74	0.619	75	0.247	70	1.460	76	0.765
Present	10	0.880	10	0.170	10	0.963	10	2.044
p-value		0.730		0.754		0.391		0.609
BRAF	84		85		80		86	
Absent	62	0.619	63	0.198	58	1.133	64	1.123
Present	22	0.905	22	0.341	22	3.709	22	0.589
p-value		0.555		0.779		0.338		0.135
RAS	83		84		79		86	
Absent	64	0.696	65	0.266	61	1.420	66	0.801
Present	19	0.219	19	0.191	18	1.010	19	1.204
p-value		0.193		0.096		0.082		0.792

* The 2-ΔΔCT method was used to quantification of miRNA expression normalized to miR-16.

Legend: *TERTp*: telomerase reverse transcriptase promoter; *BRAF*: B-Raf proto-oncogene, serine/threonine kinase; *NRAS*: NRAS proto-oncogene, GTPase; *HRAS*: HRas proto-oncogene, GTPase, and *KRAS*—*KRAS* proto-oncogene, GTPase.

Supplementary Table S7. A. Associations between miR-146b expression and clinicopathological features in Papillary Thyroid Carcinomas.

miR-146b and clinicopathological characteristics n =54 (100%)	Median*	P25 - P75*	Min - Max value*	p-value
Mean tumor size (SD) (mm) (n=54)	-	-	-	0.179
Extra thyroidal extension				0.318
Absent 42 (77.8)	27.227	3.127 - 389.557	0.016 - 6476.520	
Present 12 (22.2)	147.041	3.333 - 1402.208	0.473 - 27755.768	
Capsule invasion				**0.011**
Absent 29 (53.7)	8.733	1.131 - 119.808	0.016 - 5276,991	
Present 25 (46.3)	126.683	20.400 - 1377.380	0.473 - 27755,768	
Vascular invasion				0.270
Absent 45 (83.3)	27.945	3.119 - 448.500	0.016 - 6476.520	
Present 9 (16.7)	95.902	13.774 - 2771.874	0.473 - 27755.768	
Lymphatic invasion				0.475
Absent 48 (88.9)	29.856	3.215 - 532.402	0.016 – 6476.520	
Present 6 (11.1)	732.253	1.215 - 10022.989	0.473 – 27755.768	
Psammoma bodies				**0.019**
Absent 47 (87)	22.303	3.006 - 616.304	0.016 – 6476.520	
Present 7 (13)	160.507	61.201 - 5276.991	32.819 - 27755.768	
Calcifications				**0.010**
Absent 43 (79.6)	22.303	2.725 - 280.695	0.016 - 5268.565	
Present 11 (20.4)	160.507	32.819 - 5276.991	7.207 - 27755.768	
Lymph node metastases				0. 350
Absent 49 (90.7)	32.819	3.740 – 843.629	0.016 – 27755.768	
Present 05 (9.3)	3.070	0.968 – 746.444	0.473 – 1431.686	

* The 2-ΔΔCT method was used to quantification of miRNA expression normalized to miR-16.

Legend: P25-75: percentile 25 and percentile 75. Max-min: maximum and minimum value.

Supplementary Table S7. B. Associations between miR-221 expression and clinicopathological features in Papillary Thyroid Carcinomas.

miR-221 and clinicopathological characteristics n =67 (100%)	Median*	P25 - P75*	Min - Max value*	p-value
Mean tumor size (SD) (mm) n =67	-	-	-	0.817
Extra thyroidal extension				0.078
Absent 54 (80.6)	2.727	1.034 - 9.257	0.192 - 36.928	
Present 13 (19.4)	7.786	2.223 - 14.415	1.545 - 63.304	
Capsule invasion				**<0.001**
Absent 39 (58.2)	1,618	0.878 - 6.802	0.192 - 18.908	
Present 28 (41.8)	8.064	2.855 - 15.441	0.368 - 63.304	
Vascular invasion				**<0.001**
Absent 56 (83.6)	2.355	1.068 - 8,065	0.192 - 34.725	
Present 11 (16.4)	12.518	5.452 - 23,279	2.342 - 2.342	
Lymphatic invasion				0.078
Absent 60 (89.6)	2.900	1.240 - 1240	0.192 - 36.928	
Present 7 (10.4)	16.415	1.820 - 23.279	0.368 - 63.304	
Oncocytic component				**0.002**
Absent 58 (86.6)	2.342	1.070 - 7.972	3.006 - 616.304	
Present 9 (13.4)	12.414	8,283 - 28.598	5.452 - 63.304	
Calcifications				**0.031**
Absent 54 (80.6)	2.403	1.072 - 8.126	0.192 - 63.304	
Present 13 (19.4)	8.224	3.376 - 16.974	0.368 - 23.279	
Lymph node metastases				0.499
Absent 61 (91)	3.190	1.145 - 10.018	0.192 - 63.304	
Present 06 (9)	5.413	1,751 - 11.378	1.545 - 17.532	

* The 2-ΔΔCT method was used to quantification of miRNA expression normalized to miR-16.

Legend: P25-75: percentile 25 and percentile 75. Max-min: maximum and minimum value

Supplementary Table S7.C. Associations between miR-222 expression and clinicopathological features in Papillary Thyroid Carcinomas.

miR-222 and clinicopathological characteristics n =69 (100%)	Median*	P25 - P75*	Min - Max value*	p-value
Mean tumor size (SD) (mm) (n=69)	-	-	-	0.253
Extra thyroidal extension				**<0.001**
Absent 56 (81.2)	2.210	0.870 - 5.603	0.114 - 43,231	
Present 13 (18.8)	14.006	4.238 - 36.416	0.908 - 3006.772	
Capsule invasion				**0.005**
Absent 39 (56.5)	1.669	0.717 - 4.347	0.114 - 68.403	
Present 30 (43.5)	5.284	2.014 - 14.113	0.861 - 3006.772	
Vascular invasion				**<0.001**
Absent 57 (82.6)	2.222	0.879 - 4.806	0.114 - 68,403	
Present 12 (17.4)	10.575	7.220 - 38.986	1.392 - 3006.772	
Lymphatic invasion				**0.020**
Absent 61 (88.4)	2.325	0.902 - 8.224	0.114 - 68.403	
Present 8 (11.6)	8.172	3.840 - 36.370	1.392 - 3006.772	
Psammoma bodies				**0. 010**
Absent 58 (84)	2.251	0. 888 - 7.806	0.114 - 68.403	
Present 11 (16)	8.230	3.465 - 15.210	1,392 - 3006.772	
Calcifications				**0.006**
Absent 55 (79.7)	2.222	0.861 - 5.921	0. 114 - 68,403	
Present 14 (20.3)	7.949	3.102 - 19.672	1.392 - 3006.772	
Oncocytic component				**0.008**
Absent 50 (72.5)	1.775	0.843 - 6.496	0.114 - 3006.,772	
Present 19 (27.5)	13.211	3.673 - 32.168	2,222 - 41.556	
Inflammatory infiltrate				**0.019**
Absent 50 (72.5)	2.040	0.888 - 7.806	0.114 - 68.403	
Present 19 (27.5)	4.965	3.354 - 16.633	0.429 - 3006.772	
Tall cells				**0.017**
Absent 59 (85.5)	2.222	0.897 - 8.219	0.114 - 68,403	
Present 10 (14.5)	8.230	4.055 - 36.352	3.354 - 3006.772	
Focality				**0. 049**
Unifocal 40 (58)	3.200	1.000 - 7.669	0.114 - 68.403	
Multifocal 29 (42)	7.070	1.475 - 27.044	0.196 - 3006.772	
Lymph node metastases				0. 330
Absent 62 (89.9)	3.093	0. 951 - 12.659	0. 114 - 3006.772	
Present 07 (10.1)	4.965	1.392 - 8.675	0. 908 - 41.556	

* The 2-ΔΔCT method was used to quantification of miRNA expression normalized to miR-16.

Legend: P25-75: percentile 25 and percentile 75. Max-min: maximum and minimum value.

Supplementary Table S7. D. Associations between miR-15a expression and clinicopathological features in Papillary Thyroid Carcinomas.

miR-15a and clinicopathological characteristics n =68 (100%)	Median*	P25 - P75*	Min - Max value*	p-value
Mean tumor size (SD) (mm) (n=68)	-	-	-	0.649
Extra thyroidal extension				0.182
Absent 56 (82.4)	2.210	1.309 - 37.281	0.282 - 99.640	
Present 12 (17.6)	1.333	1.194 - 3.570	0.847- 52.086	
Capsule invasion				**0.006**
Absent 39 (57.4)	1.577	0.881 - 4.089	0.282 - 81,366	
Present 29 (42.6)	19.882	1.527- 41.928	0.712 - 99.640	
Focality				**0. 041**
Unifocal 39 (57.4)	1.867	1.249 - 37.278	0.473 - 99.640	
Multifocal 29 (42.6)	2.561	1.745 - 38.385	0.712 - 55.047	
Lymph node metastases				0. 380
Absent 61 (89.7)	2.475	1.263 - 37.257	0. 282 - 99.640	
Present 07 (10.3)	1.374	0.935 - 19.882	0. 847 - 37.705	

* The 2-ΔΔCT method was used to quantification of miRNA expression normalized to miR-16.

Legend: P25-75: percentile 25 and percentile 75. Max-min: maximum and minimum value
